# Cost-effectiveness of screening for open angle glaucoma in developed countries

**DOI:** 10.4103/0301-4738.73684

**Published:** 2011-01

**Authors:** Anja Tuulonen

**Affiliations:** Department of Ophthalmology, University of Oulu and Tampere University Hospital, Finland

**Keywords:** Cost-effectiveness, glaucoma, screening

## Abstract

As all developed countries are struggling with health care costs growing too large and too fast, the current performance and overburden of glaucoma services demand a reappraisal of current management strategies. The performance of the glaucoma care in western countries offers several opportunities to improve the simultaneous under- and over-diagnosis and treatment. Since available resources are finite, they should be targeted to produce the best eye health. There is an obvious need for prioritization of all interventions, including improving case finding. The limited evidence to date indicates that we do not have enough evidence to decide whether systematic population screening could be cost-effective in the developed world. This article gives an overview of the methods of economic evaluation and the evidence on cost-effectiveness of systematic screening for glaucoma in the developed world, need for future research and challenges related to evaluation of increasing economic literature as well as need to change behaviors on the basis of evidence.

Glaucoma is the second most common cause of visual disability in the elderly in the developed world. The performance of its current care in western countries is far from optimal and offers many opportunities to improve, e.g. all health care systems suffer from unequal access to care, large variations in the distribution of services both between and within the countries as well as problems with simultaneous under- and over-diagnosis and treatment.[1] More than half of glaucoma patients are undiagnosed,[2] half of the patients treated for glaucoma do not have the disease,[3] about 50% of glaucoma patients do not use their drops regularly (ranging from 5 to 80%)[4] and half of newly diagnosed patients found through screening studies have seen an ophthalmologist or an optometrist, but their disease was not diagnosed.[5,6]

Since we should target the available limited resources to produce the best eye health (i.e. increasing both the length and quality of “seeing years”), it is obvious that choices need to be made by prioritising all interventions, including screening, case finding, diagnostic and follow-up tests, different treatment modalities, care processes, and practices. If resources are used for one purpose (or one eye disease), they cannot be simultaneously used for something else, thus creating *opportunity* costs in terms of health benefits foregone elsewhere.[7] If and when the resources are finite, it is appropriate to ask, e.g. whether we should try to find and treat patients with undiagnosed manifest glaucoma instead of treating patients without measurable abnormalities. There are two approaches to try to find and treat those patients who are unaware of their glaucoma: either make the existing system work better by improving current opportunistic case finding, or change the system by initiating a systematic population screening program. In addition, the health care systems need to ensure enough capacity to care for the newly detected cases. The aim of this review is to evaluate what is known about the cost-effectiveness of screening for glaucoma in the developed countries and what we can learn from the so far published literature.

## Principles and Main Concepts of Economic Evaluation

The fundamental problem facing all health care systems is how to make them more cost-effective.[8] Every professional who makes decisions about individual and groups of patients is a decision-maker in health care. Our decisions should base on applications of evidence-based medicine and health care,[9] i.e. proper equitable decision-making requires high-quality, evidence-based data where we should consider: (1) who gets the services, (2) who pays for them, and (3) who gets paid for doing what–and for each category “how much”.[10] As it is, especially, the cumulative effect of small changes in clinical practices (e.g. adding new diagnostic tests or therapies) that has a massive impact on the health care budgets, clinicians need to weigh not only their benefits and risks, but should also consider the costs.[9,11,12] Today it is not anymore enough to show that an intervention is effective, it should also be cost-effective. On the other hand, by definition every cost-effective intervention is also clinically effective.

### Basic definitions (efficacy, effectiveness, and efficiency)

*Efficacy* is an outcome of intervention in ideal settings (e.g. randomized controlled trial or selected patient material at a specialist center), while *effectiveness* describes outcome in everyday practice. Efficacy is always better than effectiveness due to larger variation of “usual” patients compared to included and excluded patients in studies as well as less experienced practitioners, variability of practice patterns, etc. Therefore, although the best evidence of efficacy can be reached by randomized controlled trials, for economic evaluation the published studies so far are often “small and tight” due to their relatively small sample sizes, tight inclusion, and exclusion criteria (i.e. selected patients compared to “usual” patients), protocol-driven costs such as frequent tests and visits, as well as short follow-up considering all costs and outcomes in the course of chronic diseases [Table 1].[7]

**Table 1:** Summary of some definitions used in the text[7,18]

Intervention Any procedure carried out with a view of improving eye health of a single patient and/or the entire population – E.g. screening, case finding, diagnostic and follow-up tests, different treatments and technologies, care processes, practice patterns, etc.
Every-day practice “Usual” (unselected) patients with varying adherence and compliance to visits, tests, and treatments Practitioners with varying levels of experience, expertise and adherence to guide lines Large variability of practice patterns and access to care
Effectiveness *Outcome* of any intervention *in everyday practice* (i.e. effectiveness is worse than efficacy) – When resources and costs (= inputs) are combined to form production processes, the eye care system produces services, operations, procedures, technologies, etc. (= outputs) which are expected to improve (eye) health (= effectiveness)
Efficacy *Outcome* of any intervention *in ideal settings*, e.g. randomized controlled trial (RCT) or selected patient material at an expert center – Efficacy is better than effectiveness
Efficiency A relationship between health effects and costs (cost-effectiveness)
Productivity Technical efficiency (a relationship between outputs and inputs) – Baring in mind the limited availability of resources (inputs), productivity as such does not guarantee improved eye health unless the system is able to produce “*right things for right patients at right time in a right place*.”
Ideal study design for economic evaluation Randomized controlled trial between different alternative interventions “Usual” patients, “usual” treatment protocol, nonexpert (in addition to expert) clinical know how, long follow-up, follow-up of dropouts, and large sample size – For economic evaluation, e.g. treatment RCTs are “small and tight” due to relatively small sample sizes, tight inclusion and exclusion criteria, protocol-driven costs (frequent tests and visits) and short follow-up Measures outcome, quality of life, and costs

Economic evaluation of health care procedures and technologies is about assessing their *efficiency*, that is the produced health effects are weighed against the sacrifices or costs required attaining them. Efficiency is thus defined as a relationship between health effects and costs. Economic evaluation deals with establishing the efficiency of the *whole* treatment process compared to another treatment process.[7,9]

The economic evaluation should be made from the societal perspective, that is *all* costs (the value of all resources required by the process) are taken into account regardless of who incurs them and who pays for them. The principle in economic evaluation is to report the resources used separately from their unit costs. This helps to interpret the results of a study from one setting to another, as unit prices are known to vary by location and country. Charges should also be separated from costs since they may bear little resemblance to economic costs.[13] The charges may also change with time, e.g. the average US charge per laser trabeculoplasty in 2000 was only 40% of the highest average charge per procedure in 1989 although the technology and techniques were unchanged during the decline of reimbursement for procedure.[14]

### Types of economic analysis (cost-effectiveness, cost-utility, cost minimization, cost-benefit, and decision-analytical modeling)

When health effects are measured by simple indicators in “natural” or physical units (such as lives saved, life-years or seeing-years gained, years of blindness avoided, painless/healthy days gained), or numerous disease-specific clinical measures (for example changes in visual acuity, intraocular pressure, or visual field indices) and they are related to costs, we are speaking of *cost-effectiveness analysis*. The cost-effectiveness can only be shown in relation to a defined alternative. Thus, an intervention is never cost-effective in itself.[15] The efficiency criterion is the additional cost per additional unit of effectiveness (*incremental cost-effectiveness ratio, ICER*) [Table 2].

**Table 2:** Summary of types of economic evaluation[7,18]

Cost-effectiveness analysis Measures health effects in “natural” units or disease-specific clinical measures which are related to costs – E.g. lives saved, life-years or seeing-years gained, years of blindness avoided or changes in visual acuity, intraocular pressure, visual field indices, etc. Cost-effectiveness can only be shown in relation to a defined alternative, i.e. intervention is never cost-effective in itself. Every costeffective intervention is, however, clinically effective. – Efficiency criterion is the additional cost per additional unit of effectiveness (= *incremental cost-effectiveness ratio, ICER*) Difficulties arise if e.g. the side effects of the alternative interventions are different
Cost-utility analysis A special form of cost-effectiveness analysis (currently regarded the best method of economic evaluation) Health effects are measured in change *both in length and quality of life* Allows measuring effectiveness in terms of a change in Quality-Adjusted Life Years (QALYs) – Changes in QALYs are related to changes in costs: the efficiency criterion of cost-utility analysis is the ratio between change in costs and change in QALYs (=*incremental cost-utility ratio, ICUR*)
Quality-Adjusted Life Year (QALY) Requires the same *generic* (non-disease-specific) instrument – Produces a single index number for quality of life that reflects a plausible exchange rate between quality and length of life on a 0–1 scale (0 = death, 1 = perfect health) – E.g. the SF6, the EQ-5D (formerly the EuroQoL), 15D, etc.
Cost-minimization analysis May be used if interventions lead to virtually the same clinical outcomes, i.e. *identical outcomes of interventions at the least cost*. Identical outcomes are, however, relatively rare.
Cost–benefit analysis Health effects are measured and valued in *monetary* terms, i.e. *both the costs and benefits are measured in the same units*, e.g. whether the monetary benefits of a single intervention are greater than the monetary costs – Valuation methods of health effects in monetary terms are more or less disputable – The efficiency criterion is *cost–benefit ratio or net benefit*
Decision-analytical modeling Allows projections of long-term outcomes from short-term trial data E.g. Markov models are particularly suited for the calculation of QALYs and modeling of chronic progressive disease: – The disease is divided into various states with transition probabilities over a period of time (*cycle*) – Estimates of resource use, risks, and health outcomes are attached to the states and transitions, and the model is run over a large number of cycles – Due to parametric uncertainty probabilistic sensitivity analysis is recommended

The problem with this method often is that the indicators describe health effects inadequately and narrowly. Difficulties arise, if for example the main therapeutic effect of the alternatives to be compared is different (e.g. one may have an effect mainly on length of life, another on its quality) or if the side effects of the alternatives are different in amount or severity. Then, the comparability across alternatives is difficult, even impossible.

*Cost-utility analysis* is presently regarded as the best method of economic evaluation in health care. It is a special form of cost-effectiveness analysis in which health effects are measured in terms of change both in length and quality-of-life. These changes are aggregated into a single index number by weighting length of life with people’s “exchange rate” between quality and length of life. This “exchange rate” is elicited from population, or patients with valuation studies. This allows measuring effectiveness in terms of a change in Quality-Adjusted Life Years (QALYs). QALYs are composed in the same principle as the total points e.g. in ski jumping–points from the length of the jump (length of life) and points from its style (quality of life).[7] The total points (QALYs) can be increased by improving style (quality of life) and/or lengthening the jump (life). The changes in QALYs are then related to changes in costs. The efficiency criterion of cost-utility analysis is thus an *incremental cost-utility ratio (ICUR*, the ratio between change in costs and change in QALYs).

To be able to compare the efficiency of different interventions in terms of cost-utility for the same disease (or even different interventions for different diseases) against each other, it requires the measurement of changes in quality of life with a generic (non-disease-specific) instrument, e.g. the EQ-5D (formerly the EuroQoL), the SF6, Canadian Health Utilities Index (HUI), and 15D.[16–18] This means that one uses the same instrument for measuring quality of life regardless of what disease has brought about the changes in quality of life. In addition, the instrument must produce a single index number for quality of life that reflects a plausible exchange rate between quality and length of life on a 0–1 scale (0= death, 1= perfect health).[7]

If treatments lead to the same clinical outcomes, *cost-minimization analysis* can be used. In this approach, one is looking for the treatment alternative that produces identical clinical outcomes at the least cost. Unfortunately, the cases are relatively rare where clinical outcomes across alternatives are virtually the same.[7]

If health effects are measured and valued in monetary terms and they are weighed against costs, we are dealing with *cost-benefit analysis*. The advantage of this form of analysis is that both the costs and benefits are measured in the same units. It is then possible to examine the efficiency of even a single pharmaceutical, that is, whether its monetary benefits are greater than the monetary costs. The biggest problem of this type of analysis is the valuation of health effects in monetary terms: all valuation methods are more or less disputable. The efficiency criterion is cost–benefit ratio or net benefit.[7]

An ideal study design also for economic evaluation consists of a randomized design with measures of outcome, quality of life and costs, “usual” patients, “usual” treatment protocol, nonexpert (in addition to expert) clinical experience, long follow-up, follow-up of dropouts, and large sample size. As the length of follow-up in the clinical trials are too short for the purposes of economic evaluation, modeling studies have been undertaken making projections of long-term outcomes from short-term trial data. The use of *decision-analytical modeling* to estimate the cost-effectiveness of health care interventions is becoming widespread.[2,3,19–21] Modeling can be used to extrapolate cost and effectiveness estimates over a longer time horizon using available the epidemiological and natural history data.

Economic modeling is a relatively cheap and effective way of synthesizing existing data and evidence available on the costs and outcomes of alternative interventions. For example, Markov models have a long history of use in health care service decision-making and are particularly suited to the modeling of chronic progressive disease overtime.[19,22] In Markov modeling, the disease in question is divided into distinct states, and transition probabilities are assigned for movements between these states over a discrete time period (cycle). By attaching estimates of resource use, risks and health outcomes to the states and transitions in the model, and then running the model over a large number of cycles, it is possible to estimate the long-term costs and outcomes associated with a disease. Markov models are particularly suited for the calculation of QALYs. Cost-utility analysis on the basis of Markov models may be sensitive to parametric uncertainty. Probabilistic sensitivity analysis is, therefore, recommended especially in cases where model parameters are on the basis of limited number of observations.[3,7]

Modeling studies often criticized because of assumptions often have to be used due to inadequate evidence.[15] However, clinical and epidemiologic studies never give all relevant information, but that is no reason for not investigating what such studies can offer to assist decision-making process and future research. It appears more useful for decision makers to have some information on potential cost-effectiveness than to have no information at all. A decision is necessary regardless of whether the economic evaluation is performed.[15] A model, even if partly based on assumptions, can provide important information on potential scenarios. It is also important to realize that *all* models are wrong–including our current mental models–since they always remain imperfect and incomplete in their attempt to represent and analyze the real world.[23,24] We should, thus, not worry about whether or not to use a model, but rather which model to use.

## Cost-Effectiveness of Screening for Glaucoma

The significance of assumptions and problems of current evidence evaluating the cost-effectiveness of screening for glaucoma are highlighted in the two recent Finnish and Scottish studies which report what we know and do not about the cost-effectiveness of systematic screening for glaucoma.[2,3] These two studies agree in one major aspect: at this stage, we do not have enough proper evidence to decide whether population screening could be cost-effective in these countries.

There are several uncertainties which affect the results of both the modeling studies. Especially, the utility data in glaucoma are so far extremely limited and on the basis of cross-sectional pilot studies.[25,26] or mathematical algorithms.[27] Due to different definitions of the disease, population-based studies also show different estimates for prevalence and incidences of glaucoma in different age groups and races.[2,3] The evidence of early, moderate, and advanced stages of glaucoma is extremely limited and variable regarding how these stages are defined, how long glaucoma patients stay in each state, and what is the proportion of patients in each state.[2,3] In randomized controlled “treatment–no-treatment” trials, the progression rates have been reported for *one* eye only, that is, not per patients’ two eyes, which determines both the health-related quality of life (HRQoL) and visual disability compared to costs which are driven by the worst eye.[15] Finally, we know astonishingly little about the state we are trying to prevent, e.g. high-quality studies using severe visual impairment as an endpoint are lacking.[28]

## Needs for Future Research

The two published studies on cost-effectiveness of screening for glaucoma, however, encourage further research to study whether–although untargeted population screening might currently not be cost–effective-screening of some subgroups could be.[2,3] Their results seemingly disagreed whether screening could be cost-effective for 40 year olds compared with 60- to 75 year olds. The most probable reason for disagreeing result regarding the age was the fact that in the Finnish model also patients with diagnosis of glaucoma were screened in order to better target the treatment to the “right” subjects (= manifest glaucoma). The meaning of this finding emphasizes the great economical burden of false positives and over treatment in our health care systems.

No single (screening) test is sufficient to discriminate persons with and without glaucoma.[2] The estimates of the sensitivity and specificity of glaucoma tests show large variability[2] and are far lower than the thresholds required for *screening dominance* (= screening being less costly and more effective), i.e. specificity of 98–99% in the age group <70 years and 94-96% in the age group >70 years.[3] In addition, the majority of diagnostic studies have so far been performed on pre-selected patient populations which may lead to over-optimistic results.[29]

To study the cost-effectiveness of glaucoma screening, we would gain best evidence from a randomized trial in which one arm receives screening, the other arm current case finding and then evaluate whether systematic screening improves patient outcome, reduces glaucoma-induced visual disability and quality of life with affordable costs. Simultaneously, the diagnostic tests within the screened arm could be randomized (randomized diagnostic trial) to solve the sensitivity, specificity, and cost-effectiveness of diagnostic interventions in unscreened population (e.g. randomization between basic traditional eye examinations and modern imaging techniques and visual field tests). Such studies are lacking so far. Due to lack of a true golden standard in glaucoma diagnostics, follow-up can serve as the best possible option for golden standard.

Similarly during the follow-up, it is currently not known the “optimum” set and number of tests, i.e. *how many tests* are enough and what number represents over-testing with no additional gain incurring unnecessary expenditure. In addition, we do not know *how often* we should take the tests during the follow-up. With different examination methods, we do not know what should be the “correct” and most cost-effective threshold for initiating and intensifying treatment to prevent glaucoma-induced visual disability.[7] In addition, we should study the frequency of screening cycles. Stoutenbeek *et al*.[30] reported that the additional yield of a periodic screening program is lower than the expected from the published prevalence data. Finally, multi-eye-disease screening needs to be evaluated as to whether it would be more cost-effective than glaucoma-only screening.[31]

Further, we do not know what impact the resource utilization in glaucoma care has on an important outcome, i.e. prevention of glaucoma-Vinduced visual disability. As the current legal and cultural environments exert tremendous pressure to do more, it is important to remember that greater expenditure in the developed countries as such does not guarantee better outcomes but might sometimes even be worse.[32–34] Missing a rare–or in the case of glaucoma, a very early diagnosis or even a risk without abnormalities–may currently in the developed world be regarded worse than over-testing and over-treatment. With the shift of spectrum of detected disease, outcomes seem to improve, as newly detected cases will, in general, be milder cases (or in the case of ocular hypertension, have no manifest disease at all). This, in turn, creates stimulus to do even more. With more to do, there is also more worry, more tests, more unnecessary treatment, more mistakes–and more costs.[32]

Several papers have shown that increased costs are associated with increased disease severity.[35] From a priority setting perspective, the most important question, however, is whether the lower threshold for treatment–in spite of increase in costs-would be cost–effective in the long run in preventing visual disability compared to resource allocation for systematic screening to find patients with currently undiagnosed glaucoma. Such studies are not available at present.[36]

There are no studies on cost-utility and cost-effectiveness comparing surgical, laser, and medication therapies with each other.[36] Further research is needed to establish the efficiency of the alternative treatments for glaucoma. On the basis of very limited data comparing different therapies, it is possible that (initial) laser therapy is less expensive than (initial) medication therapy and that from a strictly economic point of view, surgery may not be cost-effective within a 3- to 4-year perspective.[36] However, with increasing follow-up (up to 8 years) the difference in costs between surgery and medication may even out. The current economic literature regarding glaucoma treatment is predominantly focused on identifying the short-term direct, particularly the precise quantification of glaucoma drug costs[35,37] and provide thus only one component of real-world costs for glaucoma.

## Other Challenges and Conclusions

So far we know very little about costs and cost-effectiveness of glaucoma screening and care. By August 2010, PubMed revealed less than 550 hits with keywords glaucoma and cost*, less than 140 hits with glaucoma and cost-effectiveness and less than 20 hits with glaucoma and cost-utility. In addition to the need for critical evaluation of *clinical* studies and application of evidence-based medicine in everyday practice, it will be even greater challenge for ophthalmologists to be able to critically evaluate the increasing number of *economic* articles. In 2007, in a sample of 1000 Finnish physicians, 80% did not know the basic concept of health economics (cost-utility) and 70% reported that their education for health economics was insufficient at medical school and during the residency program.[38] In addition, the peer reviewers as well editors need to learn a “new” discipline (health economics was born in 1950s). The fact that holds true for all scientific publications is also true with health economic papers, i.e. a published article even in a high impact journal cannot be regarded a synonym for good quality scientific evidence. This was clearly shown, e.g. in a recent health economic paper which was published in spite of a major flaw of using misleading utility values in glaucoma patients.[39]

In 2010, it is not enough to read just the abstract or the conclusion of a paper. Instead, we need to pay the most attention to materials and methods before even deciding whether to read the results. To assist critical evaluation and improve the quality and comparability of economic studies, various parties have published users’ guides for economic analysis for clinical practice.[11,12] Systematic review of the literature should be a prerequisite for granting research funding. In addition, source of research funding should also be paid attention to especially in economic papers as industry-supported reviews of drugs have been reported to show more favorable conclusions than those of Cochrane reviews.[40]

Even if the amount of high-quality economic data will increase with time, evidence as such will neither necessarily change health policies and practices[41] nor might regulators and we practitioners adopt interventions, which are demonstrably cost-effective. While not doing this, we simultaneously enhance the perception of “under-funding.”[42] Typically, we physicians practice in the fragmented, isolated tradition and do not have good enough (or are willing to utilize) administrative information available by which we could monitor: (1) what we produce in terms of activity, case mix, and outcome, (2) how we produce, i.e. what criteria we use to abandon and adopt new treatments and technologies, (3) how much we produce relative to our peers, (4) to whom we deliver care,[7] and (5) how much does it cost.

Irrespective how the health care services are financed, there is an exponentially increasing gap also in different developed countries between possibilities of diagnostic and therapeutic interventions and resources available–much more could be done than we ever can afford.[7,43] As researchers we are also a part of the problem when striving for new innovations with a hectic pace. We need to ask ourselves whether we also want to be a part of solution when all countries are struggling with health care costs growing too large and too fast, e.g. a lot of unnecessary medical costs arise from the adoption of inappropriate technologies and practice patterns.[42,43] In addition to mastering new drugs, tests, and procedures, we need to bridge the gap between “medicine” and “public health” by training medical students, residents, and all physicians to think like public health professionals.[42] There is a desperate need of future high-quality research to be able to make a decision as to which is the best, equitable, and cost-effective way to spend the money.
